# Whole Genome Sequencing of a Vietnamese Family from a Dioxin Contamination Hotspot Reveals Novel Variants in the Son with Undiagnosed Intellectual Disability

**DOI:** 10.3390/ijerph15122629

**Published:** 2018-11-23

**Authors:** Dang Ton Nguyen, Hai Ha Nguyen, Thuy Duong Nguyen, Thi Thanh Hoa Nguyen, Kaoru Nakano, Kazuhiro Maejima, Aya Sasaki-Oku, Van Ba Nguyen, Duy Bac Nguyen, Bach Quang Le, Jing Hao Wong, Tatsuhiko Tsunoda, Hidewaki Nakagawa, Akihiro Fujimoto, Van Hai Nong

**Affiliations:** 1 Institute of Genome Research, Vietnam Academy of Science and Technology, Hanoi 100000, Vietnam; dtnguyen@igr.ac.vn (D.T.N.); nguyenhaiha@igr.ac.vn (H.H.N.); tdnguyen@igr.ac.vn (T.D.N.); nthoa@igr.ac.vn (T.T.H.N.);; 2 RIKEN Center for Integrative Medical Sciences, Tokyo 108-8639, Japan; kaoru.nakano@riken.jp (K.N.); kazuhiro.maejima@riken.jp (K.M.); aya.sasaki@riken.jp (A.S-O.); tatsuhiko.tsunoda@riken.jp (T.T.); hidewaki@ims.u-tokyo.ac.jp (H.N.); 3 Vietnam Military Medical University, Ha Dong, Hanoi 100000, Vietnam; bsnguyenvanba@yahoo.com (V.B.N.); bac_hvqy@yahoo.com (D.B.N.); lebachquangdiep@gmail.com (B.Q.L.); 4 Department of Drug Discovery Medicine, Kyoto University Graduate School of Medicine, Kyoto 606-8507, Japan; jh-wong@ddm.med.kyoto-u.ac.jp; 5 Department of Medical Science Mathematics, Medical Research Institute, Tokyo Medical and Dental University, Tokyo 113-8510, Japan

**Keywords:** dioxin, intellectual disability, *ETS2*, *CENPF*, *ZNF480*

## Abstract

Although it has been a half-century since dioxin-contaminated herbicides were used to defoliate the landscape during the Vietnam War, dioxin contamination “hotspots” still remain in Vietnam. Environmental and health impacts of these hotspots need to be evaluated. Intellectual disability (ID) is one of the diseases found in the children of people exposed to the herbicides. This study aims to identify genetic alterations of a patient whose family lived in a dioxin hotspot. The patient’s father had a highly elevated dioxin concentration. He was affected with undiagnosed moderate ID. To analyze de novo mutations and genetic variations, and to identify causal gene(s) for ID, we performed whole genome sequencing (WGS) of the proband and his parents. Two de novo missense mutations were detected, each one in *ETS2* and *ZNF408* genes, respectively. Compound heterozygosity was identified in *CENPF* and *TTN* genes. Existing knowledge on the genes and bioinformatics analyses suggest that *EST2*, *ZNF408*, and *CENPF* might be promising candidates for ID causative genes.

## 1. Introduction

From 1962 to 1971, about 19 million gallons of Agent Orange (AO) and other toxic chemicals contaminated with very high concentrations of dioxin were sprayed in South Vietnam [1]. Main dioxin compounds include seven polychlorinated dibenzo dioxins (TCDDs) and 10 polychlorinated dibenzo furans (PCDFs). Among them, TCDD (2,3,7,8-tetrachlorodibenzo-p-dioxin) and PeCDD (1,2,3,7,8-pentachlorodibenzo-p-dioxin) were identified as the most toxic chemicals in laboratory animal species. Dioxin is very persistent in soil and in animal tissues (including human) [2]. 

Although it has been a half-century since the end of the Vietnam War, very high dioxin concentrations still exist in the areas of former military airbases, such as Bien Hoa, Da Nang, and Phu Cat, where the toxic chemicals were stored and used for sprays [3,4]. These hotspots of dioxin contamination have been reported in terms of soil pollutions, blood and milk concentrations, as well as health problems [5,6,7,8,9].

Dioxin is one of the most toxic compounds that can cause reproductive and developmental problems as well as cancers. In Vietnam and the United States, birth defects were observed in children of persons exposed to dioxins [10], suggesting that dioxin exposure might induce mutations of the human genome. Recently, we reported WGS of nine trios with paternal exposure to dioxin who had elevated dioxin concentrations in their sera but did not live in the hotspot areas. We found that the rates of de novo point mutations and dioxin concentrations were positively correlated [11]. However, no case in the study had been found with intellectual disability (ID). This is a neurocognitive disorder, caused by genetic and/or multiple environmental factors, usually characterized by an overall intelligence quotient (IQ) lower than 70 [12,13].

De novo mutations (DNM) occur as single nucleotide variants (SNVs), short insertions/deletions (indel), copy number variations (CNVs), or structural variations (SVs), and are shown to contribute significantly to sporadic genetic disorders, such as ID [13] and autism spectrum disorder [14], sporadic developmental diseases [15], and degenerative diseases [16].

Over the last few years, many genetic investigations of ID have been carried out using next-generation sequencing approaches [17,18]. WGS and whole exome sequencing have enabled us to identify de novo mutations in the entire genome or in the coding regions. About 1000 different genes have been reported to be associated with ID, however, the genetic etiology of up to 50% of ID cases remains unknown due to the extreme clinical and genetic heterogeneity [19].

We performed WGS of a Vietnamese family living in a dioxin contamination hotspot, where the father had a highly elevated dioxin concentration and the son was affected with an undiagnosed moderate ID, in order to identify variants which might be contributing factors in the development of the ID.

## 2. Materials and Methods

### 2.1. Subjects

A family from Bien Hoa city, Dong Nai province, Vietnam, one of dioxin contamination hotspots, was recruited through Vietnam Military Medical University and the Institute of Genome Research after obtaining informed consent. The couple lived in this location since 1975. The father was detected to have a highly elevated dioxin concentration, but the family was generally healthy, although their son, born in 1989, was affected with moderate ID. They had no other relatives with cognitive defects. The patient’s symptoms of cognitive impairment were recognized at 11 months of age. He went to school for a few years but never learned to read and write. His level of ID was in the moderate (IQ < 50) range. He was independent in self-care, such as eating, drinking, and toileting, but was unsociable. He had to be supervised when he went out. He had other signs and symptoms of health problems, such as an asymmetric body, hypotonia, and moderate twitching.

Neurological and medical assessment of the affected member was performed by clinicians of Vietnam Military Medical University. All biological samples in this study were approved by the Institutional Review Board (IRB) of Hanoi Medical University, Hanoi, Vietnam (no. 123/HMU IRB), and RIKEN, Japan. 

### 2.2. Methods

The blood samples, ~30 mL from the father and ~1–2 mL from the mother and son, were collected, frozen, and stored at −80 °C until used. For the dioxin content analysis of the father’s blood serum, potassium dichromate was added to a ~15 mL blood sample, just before delivery to the ERGO Laboratory, Hamburg, Germany, to perform high resolution mass spectrometry (HR-MS) analyses. The sample was tested for seven PCDDs and ten PCDFs according to the protocol of Schecter et al. [20].

Five hundred base pair (bp) insert libraries were prepared according to the protocol provided by Illumina and sequenced on the HiSeq2000 platform (Illumina, San Diego, CA, USA) with paired reads of 101 bp.

BWA.v0.59 [21] was used to map sequence reads to hg19/GRCh37 human reference genome. Mutation calling was performed as described previously [22]. We identified point mutations, indels, CNVs, and SVs. In addition to the previous study [22], we removed short indels that were supported by only edges of reads (10 bp from the start and end of the read) to exclude false-positive indels. We compared the mother and the proband, and the father and the proband separately. Variants found by both comparisons were considered as de novo mutation candidates. Using the depth of coverage, we detected CNVs by DNAcopy [23]; de novo CNVs were selected manually. False positive and false negative rates of de novo mutation detection were described in our recent study [11].

In addition to de novo mutations, we also searched for mutations consistent with autosomal recessive model by focusing on non-synonymous SNVs, coding indels, and variants in splice sites. Prediction of the possible impact of the amino acid substitution on the structure and function was performed using PolyPhen-2 [24], Provean [25], and SIFT [26] tools. 

## 3. Results

### 3.1. Clinical Assessment and Dioxin Level in the Father’s Serum

In this study, the total toxic equivalency value (TEQ) of dioxin and dioxin-like compounds in the serum of the father was measured as ~115 ppt while the TCDD and PeCDD were ~87 and ~12 ppt, respectively. These two very toxic congeners consisted of ~86% of the total TEQ. All of the other 15 remaining congeners constituted only ~16 ppt, or ~14% of TEQ (Table 1). 

### 3.2. Whole Genome Sequencing and Identification of Variants

The whole genomes of the three individuals (father, mother, and son) were sequenced with average ≥ 30× coverage. The total number of SNVs identified in the father, the mother, and the son were 3,436,963, 3,698,813, and 3,569,445, respectively (Table 2). Meanwhile, the total number of indels found in the father, the mother, and the son were 370,460, 403,610, and 536,126, respectively (Table 2). 

After filtering for SNVs present in the dbSNP version 138 and the 1000 Genomes database [27], the remaining SNVs, most of which should be population- or individual-specific, were considered as novel SNVs. In each of the three individuals, we identified more than 15,000 novel SNVs (Table 2). These novel SNVs are mainly located in intronic regions. In the coding region, we only identified 243, 239, and 204 SNVs in the father, the mother, and the proband, respectively. We found 63 novel indels in the father, 56 in the mother, and 45 in the proband.

### 3.3. De Novo Variants

**Figure 1:**
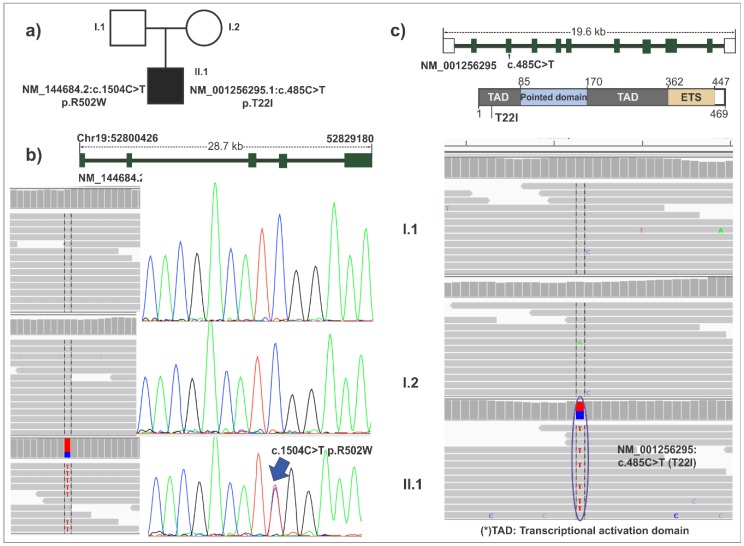
De novo heterozygous variant in *ETS2* and *ZNF480* genes. (**a**) Pedigree diagram. (**b**) De novo heterozygous variant in *ZNF480* gene. The upper part shows the structure of *ZNF480* gene, and the lower part shows the image of de novo variant NM_144684.2:c.1504C>T generated from Integrative Genomics Viewer (IGV), and the result of validation by the Sanger sequencing, respectively. (**c**) De novo heterozygous variant in *ETS2* gene. The upper part shows the structure of *EST2* gene diagram, and the lower part shows the image of de novo variant NM_001256295:c.485C>T generated from IGV viewer, and the result of validation by the Sanger sequencing, respectively.

Ninety-three de novo heterozygous variants were identified in the proband (Table S1). Of these, 76 were in non-CpG regions. The numbers of transition and transversion were 62 and 32, respectively (transition/transversion rate = 1.93). Three of the 93 de novo variants were in exonic regions, and of these, two were missense variants in *ETS2* and *ZNF480* genes (*ETS2:* NM_001256295.1:c.485C>T (p.T22I) and *ZNF480:* NM_144684.2:c.1504C>T (p.R502W)) (Figure 1, Table 3). Both variants were successfully validated using the Sanger sequencing method. One de novo deletion (NC_000008.10:g.35961275delTGGAC) in the noncoding region was identified and also validated with the Sanger sequencing method (not shown). 

### 3.4. Analysis of Germline Variations under Recessive Model

**Figure 2:**
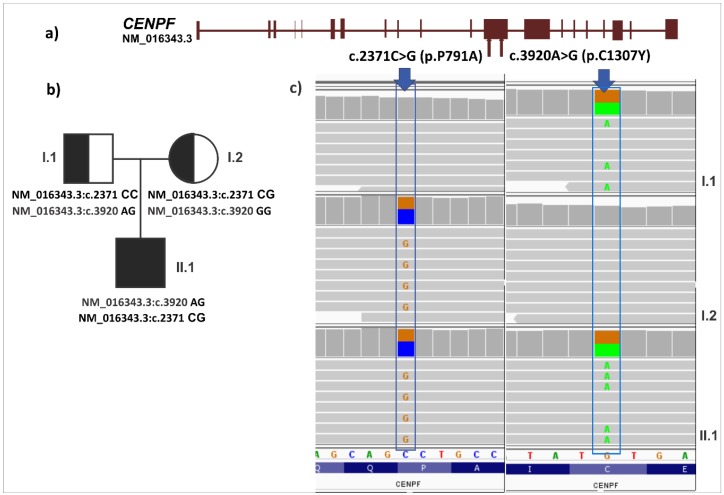
Compound heterozygous variants in the *CENPF* gene. (**a**) Structure of the *CENPF* gene diagram. (**b**) Segregation of variants. (**c**) Image of the variants at position NM_016343.3:c.2371C>G and NM_016343.3:c.3920A>G generated by IGV.

Variations were filtered under the assumption of the recessive model. Compound heterozygous variations were found in *CENPF* (p.P791A and p.C1307Y) (Figure 2) and *TTN* (p.P13274S and p.K8548N) genes (Table 3). In addition, we verified the two variants in the *CENPF* gene and were not able to find them in a total of 192 Vietnamese healthy individuals. Analysis of CNVs identified two homozygous large deletions (Table 3).

## 4. Discussion

TCDD and PeCDD are two of the most toxic chemicals of PCDDs. TCDD is widely known to come from 2,4,5-trichlorophenoxyacetic acid (2,4,5-T) herbicide, while PeCDD is believed to derive from 2,4-dichlorophenoxyacetic acid (2,4-D) herbicide, produced in the middle of 20th century [28]. From 1999 to 2001, Schecter et al. showed the TCDD blood level of people living in Bien Hoa dioxin contamination hotspot was as high as 68–413 ppt and contributed to more than 90% of the total TEQ, while the typical blood TCDD levels in the general population of Vietnam have been found to contain about 2 ppt in the South and 1 ppt in the North [5]. 

The TCDD level of the father’s blood in this study was 87 ppt and contributed to 75% of the total TEQ, which was in the range of these estimations and much higher than those of reported normal blood TCDD levels in the South Vietnam [5]. The blood sampling of the mother and son was limited, and the amounts were not suitable for dioxin measurements.

Our analysis detected 93 de novo heterozygous variants in the proband. The number of variants corresponds to a rate of 1.42 × 10^−8^ variants per nucleotide per generation which is consistent with previous reports, ranging from 1.1 × 10^−8^ to 3.8 × 10^−8^ variant per nucleotide per generation [29,30,31,32,33]. The proportion of variants in the CpG site (18/94 = 0.19) and the transition/transversion rate (62/32 = 1.9) were similar to a previous study [28].

In an attempt to identify possible causal gene(s) for ID, we analyzed genes with de novo variants, CNVs, and germline variations. De novo non-synonymous variants were found in *ETS2* and *ZNF480* genes. Compound heterozygous variants were detected in *CENPF* and *TTN* genes. The homozygous CNVs, which were hemizygous in the parents, contained *NBPF25P, KLRC1*, and *KLRC2* genes. Variants in the *TTN* gene were found with low frequency in East Asian population (0.0015 and 5.8 × 10^−5^) reported in ExAC database, while *NBPF25P* is a pseudogene. The *KLRC1* and *KLRC2* genes encode killer cell lectin-like receptor C1/2. Therefore, we considered that they are unlikely to be causal gene(s) of ID, and focused on *ETS2*, *ZNF480*, and *CENPF* genes.

The *ETS2* gene encodes a transcription factor that regulates numerous genes and is overexpressed in the brain and fibroblasts of Down syndrome (DS) individuals. *ETS2* can trans-activate the APP gene via specific Ets-binding sites in the APP promoter, resulting in the increase of beta-amyloid production in patients with DS [34]. Mutation in the *ETS2* transactivation domain located in the N-terminal part of the protein has been shown to affect its activity [35]. Our study identified a missense variant (NM_001256295.1:c.475C>T) in *ETS2*, resulting in p.T22I at the N-terminus of the protein, which might alter protein translocation. 

The *ZNF480* gene belongs to a large family of transcription factors [36], which are expressed in different parts of the human brain [37]. We identified a missense variant, NM_144684.2:c.1504C>T (p.R502W), located in the zinger finger C2H2 domain. The SIFT and Polyphen-2 software also estimated this variant to be pathogenic. A previous study found two de novo nonsense variants of *ZNF480* in schizophrenia patients, and variants in other C2H2-type zinc finger proteins have been reported to be associated with ID [18,38]. Although existing knowledge of *ZNF480* is very limited, the results suggest that *ZNF480* can be a candidate for a causal gene for ID in the patient.

Applying a recessive model, we identified a compound heterozygous variant in *CENPF*, which encodes the kinetochore protein expressed during the cell cycle [39] and in different parts of brain, as another candidate gene. A previous study identified compound heterozygous variants in *CENPF*, leading to truncated proteins, in patients with ciliopathy and microcephaly phenotypes [39]. In our study, p.P791A and p.C1307Y were found in the *CENPF* gene (Figure 2). The NM_016343.3:c.2371C>G (p.P791A) was detected with a frequency of 8.32 × 10^−6^ in the Exome Aggregation Consortium database. The variation p.C1307Y is predicted to have an effect on protein function by Polyphen-2 (score = 1.00) and Provean (score = −4.22) programs. The very low frequencies in the general populations and the predicted functional effect of the variants suggest that the *CENPF* gene is a promising candidate gene for ID.

## 5. Conclusions

In this study, we presented our comprehensive analysis of genetic variations and de novo variants in a family whose father had up to 115 TEQ ppt in his serum as a result of living in a dioxin hotspot. We identified de novo variants in *ETS2* and *ZNF480* genes and the compound heterozygous variations in *CENPF* in the son with an undiagnosed ID. Considering the functional roles of these genes and previous studies on mental diseases, they might be promising candidates for further studies performing functional analyses.

## Figures and Tables

**Table 1:** The levels of TCDD (2,3,7,8-tetrachlorodibenzo-p-dioxin), PeCDD (1,2,3,7,8-pentachlorodibenzo-p-dioxin), and other dioxin congeners in the father’s blood serum.

Congener	TEQ (ppt)
Sample amount (g)	15.236
lipid content (%)	0.177
Sample weight (lipid) (g)	0.027
Values in pg/g (ppt), lipid based	
2.3.7.8-Tetra-CDD (TCDD)	87
1.2.3.7.8-Penta-CDD (PeCDD)	12
1.2.3.4.7.8-Hexa-CDD	9.0
1.2.3.6.7.8-Hexa-CDD	33
1.2.3.7.8.9-Hexa-CDD	8.2
1.2.3.4.6.7.8-Hepta-CDD	35
OCDD	385
2.3.7.8-Tetra-CDF	3.2
1.2.3.7.8-Penta-CDF	2.8
2.3.4.7.8-Penta-CDF	9.6
1.2.3.4.7.8-Hexa-CDF	27
1.2.3.6.7.8-Hexa-CDF	15
1.2.3.7.8.9-Hexa-CDF	n.d. (3)
2.3.4.6.7.8-Hexa-CDF	8.4
1.2.3.4.6.7.8-Hepta-CDF	18
1.2.3.4.7.8.9-Hepta-CDF	n.d. (4)
OCDF	n.d. (13)
3,3’,4,4’-TCB (77)	n.d. (690)
3,4,4’,5-TCB (81)	n.d. (29)
3,3’,4,4’,5-PeCB (126)	123
3,3’,4,4’,5,5’-HxCB (169)	61
2,3,3’,4,4’-PeCB (105)	5005
2,3,4,4’,5-PeCB (114)	742
2,3’,4,4’,5-PeCB (118)	22,592
2’,3,4,4’,5-PeCB (123)	244
2,3,3’,4,4’,5-HxCB (156)	5873
2,3,3’,4,4’,5’-HxCB (157)	1579
2,3’,4,4’,5,5’-HxCB (167)	2883
2,3,3’,4,4’,5,5’-HpCB (189)	938
Total PCDDs/PCDFs	654
TEQ (World Health Organization, WHO) based on PCDD/F	115

**Table 2:** Summary of genetic variations.

	Type	Father	Mother	Proband
WGS deep coverage (x)		32.2	31.8	31.5
SNV		1,461,494	1,419,542	1,388,686
Shared with dbSNP v138		1,438,017	1,396,439	1,366,410
Shared with 1000G		6619	6963	6526
Novel variants		16,858	16,140	15,750
	Intronic	13,555	13,068	12,751
	Exonic	243	239	204
	5’UTR	54	46	38
	3’UTR	309	289	259
	ncRNA_intronic	2551	2355	2379
	ncRNA_exonic	145	140	117
	Splicing site	1	3	2
Indel		185,588	180,389	174,440
Shared with dbSNP v138		91,552	89,948	87,532
Shared with 1000G		5048	4942	4560
Novel variants		88,988	85,499	82,348
	Intronic	73,626	70,493	67,997
	Exonic	63	56	45
	5’UTR	129	114	109
	3’UTR	1850	1807	1690
	ncRNA_intronic	12,750	12,495	12,000
	ncRNA_exonic	502	479	448
	Splicing site	68	55	59
Total (SNV + Indel)		1,647,082	1,599,931	1,563,126

**Table 3:** Genes with candidate variants in the proband.

Type of Variant	Chromosomal Position	Transcript Level Position	Gene	Ref	Variant	AA Change	Provean Prediction		SIFT Prediction		Polyphen-2 Prediction	
							Prediction	Score	Prediction	Score	Prediction	Score
de novo	NC_000021.8:g.40182013	NM_001256295.1:c.47	*ETS2*	CC	CT	T22I	Not detected	Not detected	Not detected	Not detected	benign	0.055
de novo	NC_000019.9:g.52826007	NM_144684.2:c.1504	*ZNF480*	CC	CT	R502W	Deleterious	−2.74	Tolerated	0.237	probably damaging	0.999
compound heterozygosity	NC_000001.10:g.214814052	NM_016343.3:c.2371	*CENPF*	CC	CG	P791A	Neutral	−0.24	Tolerated	0.597	benign	0.001
compound heterozygosity	NC_000001.10:g.214815601	NM_016343.3:c.3920	*CENPF*	GG	GA	C1307Y	Deleterious	−4.22	Tolerated	0.087	probably damaging	1
compound heterozygosity	NC_000002.11:g.179514619	XM_005246830.1:c.907	*TTN*	GG	AG	P13274S	Deleterious	−3.53	Tolerated	0.354	benign	0.006
compound heterozygosity	NC_000002.11:g.179578790	NM_005246830.1:c.25647	*TTN*	CC	CG	K8865N	Deleterious	−3.35	Tolerated	0.091	probably damaging	0.978
homozygous deletion	NC_000001.10:g. 149040000_149195000del		*NBPF25P*		NC_000001.10:g. 149040000_149195000del (150 kb deletion)							
homozygous deletion	NC_000012.11:g.10580000_10590000del		*KLRC1, KLRC2*		NC_000012.11:g.10580000_10590000del (10 kb deletion)							

## Supplementary Materials

The following are available online at http://www.mdpi.com/1660-4601/15/12/2629/s1, Table S1: List of de novo SNVs in the proband.
