# Supplementary material for: Whole Genome Sequencing of a Vietnamese Family from a Dioxin Contamination Hotspot Reveals Novel Variants in the Son with Undiagnosed Intellectual Disability
Source: Int J Environ Res Public Health. 2018 Nov 23;15(12):2629. doi: 10.3390/ijerph15122629 (PMC6313569; doi:10.3390/ijerph15122629)
Supplement: Supplementary file 1 [file ijerph-15-02629-s001.pdf]

International Journal of
Environmental Research and Public Health

MDPI

Whole Genome Sequencing of a Vietnamese Family from a Dioxin Contamination Hotspot Reveals Novel Variants in the Son with Undiagnosed Intellectual Disability

Dang Ton Nguyen [1,2], Hai Ha Nguyen [1], Thuy Duong Nguyen [1], Thi Thanh Hoa Nguyen [1], Kaoru Nakano [2], Kazuhiro Maejima [2], Aya Sasaki-Oku [2], Van Ba Nguyen [3], Duy Bac Nguyen [3], Bach Quang Le [3], Jing Hao Wong [4], Tatsuhiko Tsunoda [2,5], Hidewaki Nakagawa [2], Akihiro Fujimoto [2,4,*] and Van Hai Nong [1,*]

1 Institute of Genome Research, Vietnam Academy of Science and Technology, Hanoi, Vietnam; dtnguyen@igr.ac.vn (D.T.N.); nguyenhaiha@igr.ac.vn (H.H.N.); tdnguyen@igr.ac.vn (T.D.N.); nthoa@igr.ac.vn (T.T.H.N.);

2 RIKEN Center for Integrative Medical Sciences, Tokyo, 108-8639, Japan; kaoru.nakano@riken.jp (K.N.); kazuhiro.maejima@riken.jp (K.M.); aya.sasaki@riken.jp (A.S-O.); tatsuhiko.tsunoda@riken.jp (T.T.); hidewaki@ims.u-tokyo.ac.jp (H.N.)

3 Vietnam Military Medical University, Ha Dong, Hanoi, Vietnam; bsnguyenvanba@yahoo.com (V.B.N.); bac_hvqy@yahoo.com (D.B.N.); lebachquangdiep@gmail.com (B.Q.L.)

4 Department of Drug Discovery Medicine, Kyoto University Graduate School of Medicine, Kyoto, 606-8507, Japan; jh-wong@ddm.med.kyoto-u.ac.jp

5 Department of Medical Science Mathematics, Medical Research Institute, Tokyo Medical and Dental University, Tokyo, 113-8510, Japan; tatsuhiko.tsunoda@riken.jp

* Correspondences: fujimoto@ddm.med.kyoto-u.ac.jp (A.F.); vhnong@igr.ac.vn (V.H.N.)

Table S1. List of de novo SNVs in the proband.

Chr	Position (Hg19)	Ref	Genotype	CpG	Transversion/Transition	Gene_region	Gene_symbol
8	38953881	T	AA	0	Transversion	intronic	*ADAM9*
5	159360230	G	AG	0	Transition	intronic	*ADRA1B*
15	44621257	G	AG	0	Transition	intronic	*CASC4*
4	91413165	G	AG	1	Transition	intronic	*CCSER1*
19	52826007	C	CT	0	Transition	intronic	*ZNF480*
5	122723763	T	CC	0	Transition	intronic	*CEP120*
16	12783269	T	GT	0	Transversion	intronic	*CPPED1*
21	40182023	C	CT	0	Transition	exonic	*ETS2*
21	33805918	C	AC	0	Transversion	intronic	*EVA1C*
15	29592703	T	CT	0	Transition	intronic	*FAM189A1*
9	128026834	A	AG	0	Transition	intronic	*GAPVD1*
19	19602403	A	AG	0	Transition	intronic	*GATAD2A*
1	210753987	T	CT	0	Transition	intronic	*HHAT*
16	25939395	C	CT	1	Transition	intronic	*HS3ST4*
7	110574326	A	AG	0	Transition	intronic	*IMMP2L*
2	48928665	T	GT	0	Transversion	intronic	*LHCGR*
12	25642875	C	CG	0	Transversion	intronic	*LMNTD1*
2	102348244	G	GT	0	Transversion	intronic	*MAP4K4*
6	150474569	C	CT	1	Transition	intronic	*PPP1R14C*
7	83233199	G	GT	0	Transversion	intronic	*SEMA3E*
9	38011043	C	CT	1	Transition	intronic	*SHB*
19	52826007	C	CT	1	Transition	exonic	*ZNF480*
3	66152636	C	CT	1	Transition	intronic	*SLC25A26*
6	155492346	C	CG	0	Transversion	intronic	*TIAM2*
14	76286442	G	GT	1	Transversion	exonic	*TTLL5*
11	122585803	A	AC	0	Transversion	intronic	*UBASH3B*
19	17372237	C	CT	1	Transition	intronic	*USHBP1*
1	108386014	T	AT	0	Transversion	intronic	*VAV3*
1	182028259	C	CT	1	Transition	intronic	*ZNF648*
1	23538156	T	AT	0	Transversion		

1	46249721	T	GT	0	Transversion
2	44237622	C	CG	0	Transversion
2	51453535	T	AT	0	Transversion
2	139600915	T	CT	0	Transition
2	169306454	C	CT	0	Transition
2	199164696	G	AG	0	Transition
3	5702434	A	AG	0	Transition
3	33273790	G	AG	1	Transition
3	59078789	A	AG	0	Transition
3	89060999	A	AC	0	Transversion
3	89061001	G	AG	0	Transition
3	89544369	T	CC	0	Transition
3	115257101	G	AG	0	Transition
3	139464120	C	AC	0	Transversion
3	162994573	C	AC	0	Transversion
4	8657803	G	AG	1	Transition
4	116826844	G	GT	0	Transversion
5	8031502	G	AG	0	Transition
5	71837052	G	AG	0	Transition
5	143972308	C	CT	0	Transition
5	151582484	T	CT	0	Transition
5	154481138	C	AC	0	Transversion
5	154481139	T	GT	0	Transversion
6	14683612	A	AT	0	Transversion
6	84207537	A	AG	0	Transition
6	120503506	G	AG	0	Transition
6	158122671	G	AG	1	Transition
7	14111847	T	CT	0	Transition
7	17545963	C	CT	1	Transition
7	38403767	A	AG	0	Transition
7	54666477	A	AC	0	Transversion
7	151026041	G	CG	0	Transversion
8	122004586	T	CT	0	Transition
8	130091717	G	AG	0	Transition

9	138428950	C	CT	0	Transition
10	16448221	C	CT	1	Transition
10	27233572	G	AG	1	Transition
10	59937141	T	CT	0	Transition
10	80693834	C	CT	1	Transition
11	57992413	A	AG	0	Transition
12	27005234	A	AT	0	Transversion
12	34491009	C	CT	1	Transition
13	87921799	T	CT	0	Transition
13	90117960	A	AG	0	Transition
14	19823638	C	CG	0	Transversion
14	82359410	A	AG	0	Transition
14	95196582	G	AG	0	Transition
14	104872678	C	CT	0	Transition
14	106267695	A	AG	0	Transition
14	106267696	C	AC	0	Transversion
15	78199077	G	GT	0	Transversion
15	78199078	G	AG	0	Transition
16	33934955	G	AG	1	Transition
17	4064193	C	CT	0	Transition
17	18551512	C	AC	0	Transversion
17	29072909	C	CT	0	Transition
17	33546294	G	GT	0	Transversion
17	33546295	C	CT	0	Transition
17	46718792	C	AC	0	Transversion
19	39733123	C	CT	0	Transition
22	17366628	A	AG	0	Transition
X	22672879	C	TT	0	Transition
X	111721362	A	AT	0	Transversion
X	111721363	A	AG	0	Transition
